# Considering choline as methionine precursor, lipoproteins transporter, hepatic promoter and antioxidant agent in dairy cows

**DOI:** 10.1186/s13568-017-0513-z

**Published:** 2017-11-25

**Authors:** Imtiaz Hussain Raja Abbasi, Farzana Abbasi, Rab N. Soomro, Mohamed E. Abd El-Hack, Mervat A. Abdel-Latif, Wen Li, Ren Hao, Feifei Sun, Bello M. Bodinga, Khawar Hayat, Junhu Yao, Yangchun Cao

**Affiliations:** 1 Department of Animal Nutrition and Feed Science, College of Animal Science and Technology, Northwest A&F University, Yangling, 712100 Shaanxi People’s Republic of China; 2 School of Life Science and Engineering, Southwest University of Science and Technology, Mianyang, 621010 Sichuan People’s Republic of China; 3 Department of Poultry, Faculty of Agriculture, Zagazig University, Zagazig, 44511 Egypt; 4 Department of Nutrition and Clinical Nutrition, Faculty of Veterinary Medicine, Damanhour University, Damanhour, 22516 Egypt

**Keywords:** Choline, Fatty liver, Dairy cows, Production, Transition period

## Abstract

During the transition period, fatty liver syndrome may be caused in cows undergo negative energy balance, ketosis or hypocalcemia, retained placenta or mastitis problems. During the transition stage, movement of non-esterified fatty acids (NEFA) increases into blood which declines the hepatic metabolism or reproduction and consequently, lactation performance of dairy cows deteriorates. Most of studies documented that, choline is an essential nutrient which plays a key role to decrease fatty liver, NEFA proportion, improve synthesis of phosphatidylcholine, maintain lactation or physiological function and work as anti-oxidant in the transition period of dairy cows. Also, it has a role in the regulation of homocysteine absorption through betaine metabolite which significantly improves plasma α-tocopherol and interaction among choline, methionine and vitamin E. Many studies reported that, supplementation of rumen protected form of choline during transition time is a sustainable method as rumen protected choline (RPC) perform diverse functions like, increase glucose level or energy balance, fertility or milk production, methyl group metabolism, or signaling of cell methionine expansion or methylation reactions, neurotransmitter synthesis or betaine methylation, increase transport of lipids or lipoproteins efficiency and reduce NEFA or triacylglycerol, clinical or sub clinical mastitis and general morbidity in the transition dairy cows. The purpose of this review is that to elucidate the choline importance and functions in the transition period of dairy cows and deal all morbidity during transition or lactation period. Furthermore, further work is needed to conduct more studies on RPC requirements in dairy cows ration under different feeding conditions and also to elucidate the genetic and molecular mechanisms of choline in ruminants industry.

## Introduction

Feed formulation technology, ingredients concentration or nutrient requirements, according to need and stage of animals play an important role in the production and health of ruminants. Excessive or unbalanced amount of nutrients or ingredients may cause disease, reduce dairy performance and increase the environmental pollution. Cows are suffering from different physiological variation throughout the transition period i.e. reduction of dry matter intake, go into negative energy or metabolizable protein balance, increase the demand of high quality nutrients to sustain growth of fetus and lactation performance, development of ketosis, hypocalcemia, clinical mastitis and fatty liver signs (Overton and Waldron 2004; Esposito et al. 2014; Itle et al. 2015; Sun et al. 2016). Through the transition stage, stress and hormonal variations enhanced non-esterified fatty acids (NEFA) into blood to overcome negative energy balance in transition cows. NEFA oxidation occurs in the liver in order to generate energy and some will renovate into triglyceride (TG) and mobilize in the form of very low-density lipoprotein (VLDL) (Pinotti et al. 2003). But, some of the NEFA is partially oxidized in the liver and consequently increased ketone bodies in the blood and export of TG through VLDL become limited. Furthermore, in pre-or early lactation cows peroxidation ratio of lipid was increased (Castillo et al. 2005) but serum α- tocopherol was reduced (LeBlanc et al. 2004) and then, oxidative stress at higher levels which decreased the health of dairy cows and their productive performance (LeBlanc et al. 2004). Choline also works as an antioxidant in transition cows, it has antioxidant properties that protect cell and reduce the free radical production which damage liver cells (Elsawy et al. 2014). During the transition period, supplementation of RPC and rumen protected methionine (RPM) alleviate the oxidative stress throughout the period (Wu et al. 2013) and increase the immune response through its antioxidant activities on immune organs (Wua et al. 2014; Sun et al. 2016). Choline [(CH_3_)_3_N + (CH_2_)_2_OHX)] also called as trimethyl ethanolamine categorized as B-complex vitamin, but this is not satisfactory definition or category of choline (Zeisel and Da Costa 2009). Because choline is endogenously synthesized agent and its deficiency syndrome is very difficult to be recognized in ruminant, because it has complex interaction with methionine, folic acid and vitamin B_12_. Sometimes, when methionine and folates deficient diet offered to ruminants, the choline works as an essential nutrient for ruminants (Zeisel 2000). Supplementation of rumen unprotected form of choline is quickly degraded by rumen microbes and the remaining quantity can’t reach to the level for absorption or maintaining further functions. So, rumen protected choline must be supplemented in the rations of cows; to continue choline dependent key mechanisms like, sustaining of animal metabolism, sparing of methyl group and remethylation of homocysteine through betaine metabolite (Esposito et al. 2014; Itle et al. 2015). The present review article aimed to highlight the choline functions and importance in the transition period of dairy cows and deal all morbidity during transition or lactation period.

### Physiological functions of choline and its important action in cell

#### Choline metabolites and their key role in cells

Choline is categorized as an essential nutrient in dairy ruminants but their logic mechanisms are still challenging and it transformed into several metabolite forms in cells for optimal growth or performance in addition to ideal health of cows (Phillips 2012). Phosphatidylcholine (PC), lysophosphatidylcholine (LPC), free choline (Cho) and sphingomyelin (SM) are the key constituents as choline containing lipid soluble metabolite for all cell membranes and have an important role in the cell signaling and lipid metabolism (Jiang et al. 2014). Choline or its metabolites are important to maintain the important physiological functions i.e. cell membrane structural veracity and signaling roles, synthesis of acetyl choline (neurotransmission) and methylation to transfer methyl group through it betaine metabolite to synthesize S-adenosylmethionine (SAM) pathway (Glier et al. 2014). Choline metabolites play a vital role in the mammalian biological development particularly; its supplementation during the transition period increases the metabolism of lipids in the hepatic cells (Veth et al. 2016). Cho, acetylcholine (ACho), glycerol phosphorus choline (GPCho), betaine (Bet), and phosphorus choline (PCho) as water-solubles serving as basic neurotransmitters in the autonomic nervous system with ACho (Cheng et al. 2008; Artegoitia et al. 2014). However, during mitochondrial oxidation of Cho, form Bet with GPCho are produced and act as organic osmolytes (Jiang et al. 2014). Furthermore, Bet undergoes oxidation in order to release methyl groups for translation of homocysteine and generation of methionine (Eklund et al. 2005; Combs 2012). Because of the complex function or action of choline and its metabolites in the cell, further research is still needed to find out the individual effects of choline metabolites on the blood plasma, milk production or composition during pre-or post-parturient period and their effect on hepatic cells.

#### Betaine as transmethylation agent

Betaine is the most vital choline metabolite (Betaine) which is important for synthesis of creatine, carnitine or methionine by methyl group donation through transmethylation pathway into cell (Eklund et al. 2005). Most of dietary products are rich sources of phosphatidylcholine, approximately contain 13% Cho by weight (Koc et al. 2002). Supplementation of choline in the ration can significantly affects the re-methylation activity of Bet, although, Bet can’t go into reverse reaction to convert back into choline but it takes a part when Cho is not abundant to maintain re-methylation reaction for homocysteine synthesis (Siljander et al. 2003). Ration supplemented with Bet can modulate methylation through homocysteine methyltransferase reaction, which regulate *S*-adenosylhomocysteine and *S*-adenosyl methionine in the cell and also improve epigenetic mechanism or methylation of DNA in the cell, availability of choline or betaine in the ration with other methyl donor group can significantly influence the methylation reactions (Zeisel 2017). Furthermore, methyl-tetrahydrofolate and betaine actively participate in the biosynthesis methylation of homocysteine to methionine (Zeisel 2017).

#### Labile role of choline and methionine

**Fig. 1:**
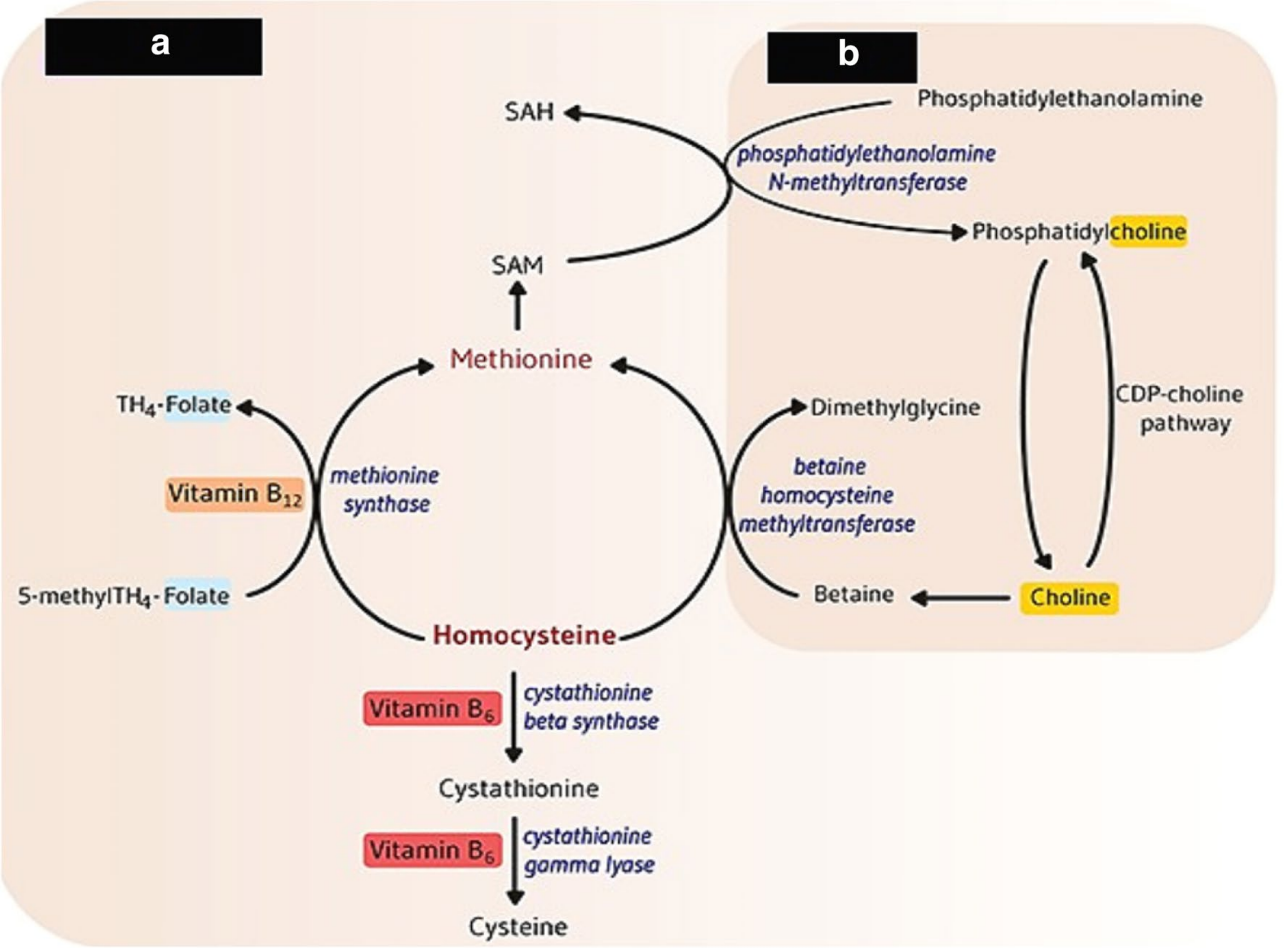
**a** Methylation reaction pathway of homocysteine, to form essential amino acid methionine and another choline metabolite betaine, dependent pathway as methyl donor for methylation of homocysteine for methionine formation and **b** demonstrated that methionine as a universal donor of *S*-adenosyl methionine (SAM) which contribute to convert phosphatidylethanolamine into phosphatidylcholine to generates choline

**Fig. 2:**
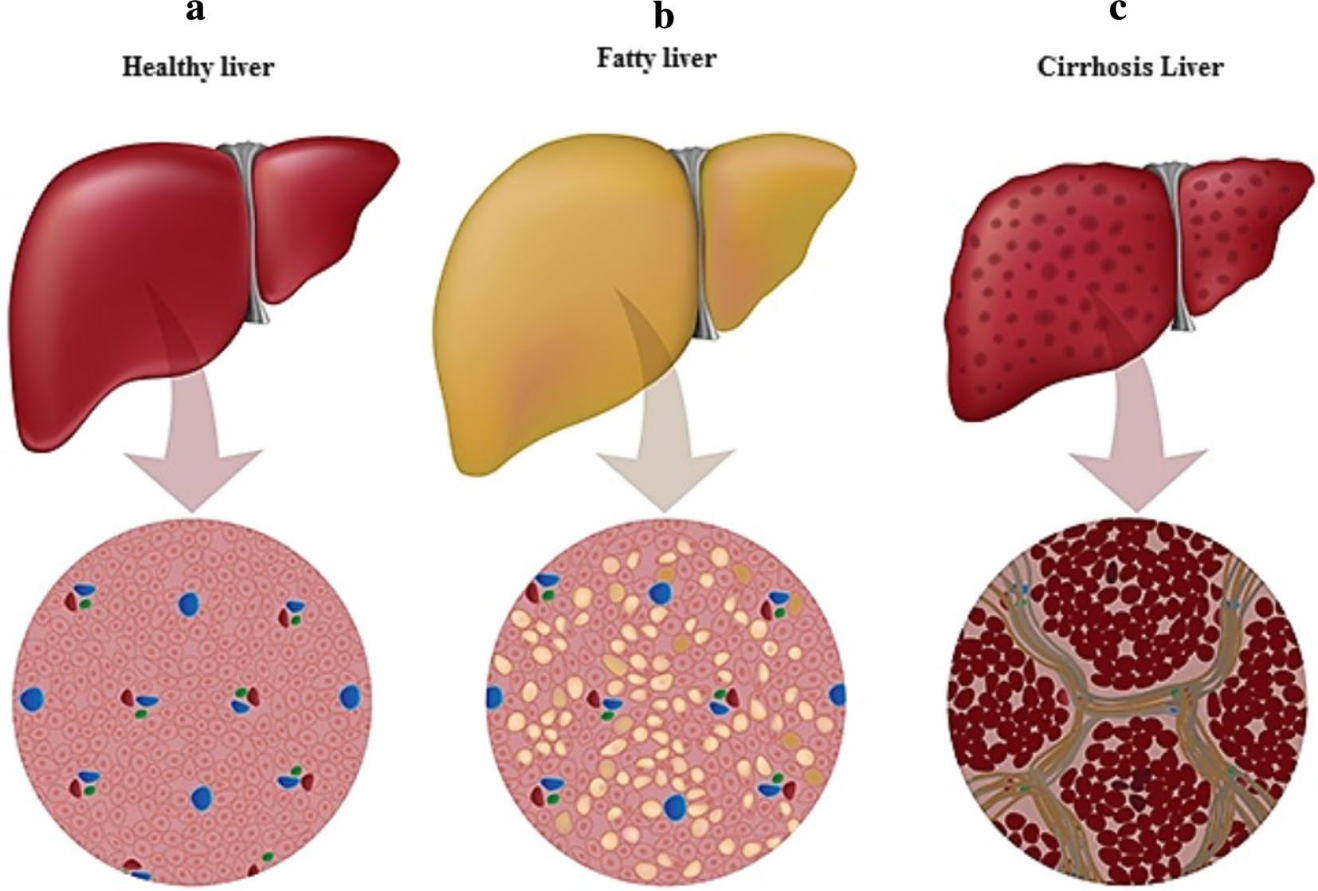
**a** Normal stage of liver. This figure shows the normal stage of liver, **b** abnormal fat and cholesterol accumulate as fatty liver, **c** as choline deficiency results in impaired VLDL secretion and accumulation of excessive fat in the liver ultimately cirrhosis condition developed

Methionine (Met) is considered as a top limiting amino acid (AA) for milk production and it is a good opportunity to supplement rumen undegraded form of methionine in the ration of dairy cows typically in order to improve milk or milk protein production, sustain contribution of SAM molecules for choline synthesis and enhance VLDL production to reduce ketones body during the early lactation period in dairy ruminants (Overton et al. 1998). In addition, both of Met and Cho are important nutrients as methyl moiety providers in ruminants. Met works as a methyl donor to endorse de novo synthesis of Cho and supplementation of Cho contributes to spare betaine metabolite for generation of sulphur containing AA (Met) as to support animal performance (Ardalan et al. 2011). Actually, Cho is a donor of Bet and Bet provide methyl group for conversion of homocysteine to provide Met which require 5 methyl tetrahydrofolate through betaine homocysteine methyl transferase enzyme (Zeisel and Corbin 2012). Then homocysteine undergoes further catabolism mechanism to produce cysteine through vitamin B_6_ transsulfuration dependent pathway (Fig. 1) or sometimes remethylated to methionine. But the quantity of folate, and Cho in blood and liver significantly affect the homocysteine regulation pathway (Leach et al. 2014). Vitamin B complex group (riboflavin, B_6_, B_12_ and folate) and Cho are compulsory for both AA and nucleic acids metabolism and take an important role in the synthesis of SAM as universal donor from Met. For methylation reaction, SAM contributes three molecules that changed phosphatidylethanolamine into phosphatidylcholine by phosphatidylethanolamine *N* methyl transferase enzyme, then further phosphatidylcholine generates choline by the action of phospholipases through cytidine diphospho choline pathway (Fig. 2) and becomes *S*-adenosylhomocysteine which further converted into homocysteine. Once homocysteine is formed, it will be metabolized into cysteine through transsulfuration vitamin B_6_ dependent pathway (Fig. 1) (Osorio et al. 2014).

### Choline impacts on NEFA production and hepatic function in transition cows

#### Non-esterified fatty acids as an energy indicator of transition cows

During the transition period, the traditional management of dairy farm provides cows stress free environment, which directly reduce mobilization or circulation of NEFA from the adipose tissue; NEFA concentration directly reflect mobilization of adipose tissue, these fatty acids circulate in the blood to maintain energy balance during the transition period (McNamara 1991). Calving is stressful condition in which NEFA and hydroxyl butyric acids concentrations were increased which are specific to negative energy balance and then, reduction of milk yield, development of fatty liver syndrome in addition to postpartum reproductive problems in cows (Grummer 2008). During the transition period, high producing cows have rapid transportation of fatty acids from adipose tissue to maintain energy balance resulting in circulation of excess NEFA in the blood which impair the hepatic function and animal performance with increase in choline metabolism (Karimian et al. 2015). Through stress, adipocytes are lipase sensitive, and the deposited triacylglycerol is broken down into glycerol and non-esterified fatty acids or free fatty acids which transported into liver, if continuous NEFA mobilization increase towards liver which directly causes hepatic inflammation or hepatic steatosis and the liver function decreased (Maison et al. 2000; Zhang et al. 2005). Continuous and excessive mobilization of the stored fatty acids from adipose tissue is a sign of low dietary energy which increase burden on liver with low hepatic fatty acid oxidation rate and low mobilization rate of VLDL (Grummer 1993). Furthermore, high uptake of NEFA by hepatic cell consequently develop hepatic lipidosis condition of cows and alleviate the capacity of detoxifying ammonia into urea which reduces gluconeogenic capacity from propionate fatty acid (Strang et al. 1998). Liver cell capacity fully engaged to esterify mobilized NEFA into TAG (Litherland et al. 2011). Excessive overload of NEFA accelerated the oxidation level of liver cells and secretion of esterified fatty acids (or TAG) in the form of VLDL and finally TG accumulation will be start and liver will go into impaired condition, fatty liver syndrome or cirrhosis condition will be developed and cows’ health or performance will be down every day (Drackley et al. 2005). To control this situation, increase production of VLDL or lipoprotein in the transition cows through rumen protected choline (RPC) supplementation which has a good opportunity, to alleviate fatty liver disorder, reduce ketosis in lactating cows and improve milk composition or yield (Reynolds et al. 2003; Castillo et al. 2005; Cooke et al. 2007).

#### The supplemented RPC affects the health and fatty liver syndrome

Fatty liver is a general sign during the transition period of dairy cows. Several steps were taken to reduce this syndrome, rumen protected choline supplementation is one of the best steps to export hepatic fat by lipoprotein pathway and improved performance of dairy cows (Zom et al. 2011; Ardalan et al. 2011; Elek et al. 2013). Choline has significant effects on general function of animal body particularly on the immunity of liver cells. During calving, hormonal variations and negative energy balance during transition or following early lactation are significant condition of higher liver uptake of NEFA which cause overload on the liver to metabolize the esterified fatty acids (Goselink et al. 2012). Feeding ration containing deficient ratio of both Cho and Met leads to decrease of vitamin C or α or γ tocopherol production which causes infection of epithelium, degeneration of muscle and damage of hepatic tissue with decrease of VLDL hepatic transportation (Henning et al. 1997). The amount of dietary fat or cholesterol affect the production of NEFA during stress condition which mobilized into hepatic cells through lipoprotein termed as chylomicrons pathway; then these fats or cholesterol wrapped named as very low-density lipoprotein (VLDL) and carried to liver through blood stream, but VLDL assembly or secretion from hepatic cell need phosphatidylcholine (Noga and Vance 2003). If phosphatidylcholine is not in abundant amount, the impaired VLDL secretion will start and accumulation of fat or cholesterol in hepatic cells will start and then fatty liver condition will develop, finally liver cells undergo steatosis and cirrhosis (Noga et al. 2002). Although concentration of foliate significantly increases demand of Cho derivative and Bet; if the ration is deficient in dietary foliate and Cho, the de novo synthesis also does not maintain body requirement of choline. Conversely, VLDL, Bet or Met balance undergoes stress and liver performance will go down (Noga et al. 2002). Choline deficiency showed atherosclerosis sign or liver function disorder and increase the quantity of liver enzyme alanine amino transferase (MIC 2017). In addition, its deficiency elevates the homocysteine level and raise the risk of dystocia or retained placenta, low fetus weight and promote inflammation of hepatic cells or cause lipid metabolism disorder, increase oxidative stress or reactive oxygen species (Zeisel and Da Costa 2009; Sun et al. 2016). Most species showed fatty liver syndrome due to dietary Cho deficiency, which is developed due to deposition of fat or triacylglycerol which decrease phosphatidylcholine, conversely, VLDL synthesis or secretion will go in termination pathway (Jiang et al. 2014). Transition period of dairy ruminants categorized as low or high fatty liver period (Bobe et al. 2004), supplementation of RPC decline deposition of triacylglycerol and increase genes expression which involved in VLDL mobilization (Zom et al. 2011). More recent studies reported that, basic bovine hepatocytes improve VLDL transportation when incubated with Cho chloride (Goselink et al. 2013).

### RPC applications and their effects on performance of cows

#### Effects of supplemented RPC on hematology and reproductive performance of cows

During the transition period, cows undergoes many physiological changes, production and metabolism of NEFA is a big challenge and the total plasma bilirubin (TBIL) concentration in the blood shows liver lipidosis conditions or liver dysfunction, during postpartum period, the concentration of TBIL of cows were found significantly high (Bionaz et al. 2007; Sejersen et al. 2012). Supplementations of RPMet and RPC particularly in the transition cows improved the hepatic function, and decreased the concentration of TBIL which showed as the sign of healthy liver status (Sejersen et al. 2012; Sun et al. 2016). In dairy cows, metabolism of free radicals remains under homeostatic condition, but in transition period, due to excessive stress, production of higher NEFA concentration and their oxidation at the hepatic cell, increase reactive oxygen species (ROS) which result in oxidative stress (Bionaz et al. 2007; Turk et al. 2013). During the transition period, supplementation of both RPC and RPMet, in plasma increases the total antioxidant capacity (T-AOC) and vitamin E concentration, but malondialdehyde content was decreased. (Sun et al. 2016). Furthermore, dietary RPC supplementation in cows had significant effects a increased serum glucose and cholesterol concentration and reduced triglyceride, NEFA and urea concentration (Soltan et al. 2012) but decreased plasma cholesterol, triglycerides percent, and glucose (Piepenbrink and Overton 2003). On the other hand, some contrast studies report that, supplementation of RPC found no significant effect on NEFA, glucose, total protein, albumin, globulin urea- N and β hydroxybutyric acid (BHBA), because there was no variation in adipose tissue mobilization or production of BHBA in the hepatic cell was found during RPC supplementation (Zahra et al. 2006; Guretzky et al. 2006). Many studies noted that, the incidence of reproductive disorder was increased during the transition period increased due to excessive mobilization of adipose tissue for synthesis milk fat, and decline the development of follicle which indicates low fertility. However, supplementation of RPC had no significant effects on pregnancy, insemination, per conception ratio and estrus cycle compared to control (Ardalan et al. 2011; Lima et al. 2012). Moreover, ration supplemented with RPC and RPMet decreases overall frequency of heath disorder during the transition period (Xu et al. 1998), compared to control group cows supplemented with RPC cows calving twins (Guretzky et al. 2006).

#### Impact of supplemented RPC on milk yield and properties of cows

Both quantity and quality of milk are the most important traits of ruminants industry. Supplementation of RPC can increase both traits due to higher digestibility, improve VFA production, reduce NH_3_–N or ketosis metabolic disorder and decrease fatty liver syndrome (Baldi and Pinnotti 2006). Further intestinal Cho supplementation improves milk performance in dairy cows about 7% more than control (Ardalan et al. 2011). Many studies reported that, supplementation of RPC increased the tendency of milk production, and fat corrected milk in lactating dairy cows (Lima et al. 2007). Cho supplementation works as a significant lipotropic agent in dairy ruminants, as it reduces excessive fat accumulation in the hepatic cells with significant and increase in milk yield, fat, lactose, solid not fat, total solid and protein yield (Zom et al. 2011; Leiva et al. 2015). Milk production, fat and protein were improved when ration supplemented with RPC and RPMet during transition period of cows (Osorio et al. 2013; Sun et al. 2016). Although, some studies reported that supplementation of RPC significantly can’t improve milk yield or fat corrected milk (Pinotti et al. 2003; Pineda and Cardosa 2015). RPC supplementation in dairy cows could not have significant effects on milk fat, protein, lactose, SNF, total solids, yield and milk urea nitrogen concentration compared to control (Xu et al. 2006; Zom et al. 2011). Supplementation of RPC had no positive effects on milk lactose and other milk components (Hartwell et al. 2000; Guretzky et al. 2006; Baldi et al. 2011). However, the experimental design (Zahra et al. 2006) supplementation of other diets (Davidson et al. 2008) application methods (Ardalan et al. 2010) animals breed (Sales et al. 2010) quantity of supplements, length of experiment or duration and phase of lactation of dairy cows significantly affect the RPC supplementation and milk production or milk properties (Guretzky et al. 2006). Milk yield, protin, fat and fat corrected milk were increased in Alfalfa and maize silage ration supplemented with RPC as (60 g/day) (Ardalan et al. 2011). Cows supplemented with RPC had significant increase in milk production when compared with cows fed other diets (Davidson et al. 2008).Multiparous Holstein cows supplemented RPC 5 an 100 g cow/day for 21 pre-parturition and 45 post parturition duration had significant increase in milk fat, protein and total solid also improved haptoglobin and insulin with milk-composition compared with control (Leiva et al. 2015). Primiparous and multiparous Holsteins after lactation for 8 week fed maize silage supplemented with RPC 56 g/day improved milk yield performance of dairy cows (Zahra et al. 2006). Meta-analysis specified that, RPC supplementation during transition period to cows increased milk yield, fat, protein, because of methylation or methionine sparing, lower liver TGA and improved gluconeogenesis process by hepatic cell (Sales et al. 2010; Goselink et al. 2012) (Table 1).

**Table 1:** Summary of different studies supplemented RPC and their effect on milk production, fat and protein % of dairy cows

References	Lactation stage pre-and postpartum days		RPC (g/day) supplement	Milk yield (kg/day)	Fat yield %	Protein yield %
	Pre	Post				
Veth et al. (2016)	–	14	0	31.30	4.04	3.46
			12.5	32.00	4.03	3.47
			25	31.39	4.04	3.42
Sun et al. (2016)	21	21	0	–	3.28	3.05
			15	–	3.44	3.19
Leiva et al. (2015)	21	45	0	30.6	3.29	3.17
			50+	29.1	3.51	3.32
			100++	–	–	–
Ardalan et al. (2011)	28	70	0	31.70	3.30	3.13
			60	34.60	3.35	3.11
Davidson et al. (2008)	21	90	0	27.90	2.97	2.60
			40	27.50	2.93	2.68
Piepenbrink and Overton (2003)			0	40.00	4.02	2.97
	21	63	45	43.30	4.21	3.02
			60	39.90	4.00	3.01
			75	41.00	4.29	3.00
Pinotti et al. (2003)	14	30	0	28.50	3.24	3.11
			20	31.40	3.36	3.05

–, not reported; +, supplemented before calving; ++, supplemented after calving

### Choline metabolism and dietary requirements for dairy cows

Dietary requirements of choline and its importance were recognized in, swine, poultry and fish but till now, many studies under investigation and try to establish the requirements and its important functions in dairy cows during the transition period (NRC 2001). Dietary choline is easily degraded through rumen microorganism and does not maintain the balance or requirements of ruminants so, they must be supplemented with rumen protected choline which bypass rumen degradation process and carry out large amount for absorption at the small intestine (Cheng et al. 2008). Furthermore, due to autonomous processes of absorption of choline metabolites at the small intestine, it makes engaged pathway to understand free choline absorption and supplemented RPC absorption through small intestine and establishes the accurate dietary requirements of choline for dairy ruminants (Cheng et al. 2008). Because absorption pathway of choline is carrier mediated transport pathway and saturable substrate specific at low concentration and passive diffusion mechanism at high concentration (Sheard and Zeisel 1986). Also, choline is absorbed via enterocytes and passes through basolateral membrane mechanism into hepatic portal system (Cheng et al. 2008; Jiang et al. 2014). During periparturient duration supplementation of RPC to cows reverses the deficiency of choline and declines the accumulation of triacylglycerol in the hepatic cells (Zom et al. 2011; Elek et al. 2013). Recently, one study reported that supplementation of RPC 12.5 g/day reduced the concentration of triacylglycerol by one-third during first 6 weeks of lactation period and improved hepatic performance (Zom et al. 2011). Supplementation of choline ion 18.7 and 37.3 g/day as RPC at the stage of pre-and postpartum, reduces liver triacylglycerol by 66% (Elek et al. 2013). The amount of supplemented RPC is an important agenda for future establishing dietary requirements for ruminants; further research focused on how much RPC should be given during the transition period. In previous research broad ranges from (6 to 45 g/day) of choline chloride has been studied in the transition dairy cows. Although, better response was noted on milk and milk fat or protein and liver fatty syndrome or triacylglycerol which were decreased when RPC was supplemented from 12 to 20 g/day to dairy ruminants (Hartwell et al. 2000; Piepenbrink and Overton 2003; Zom et al. 2011; Elek et al. 2013; Itle et al. 2015).

## Conclusion

The role of choline in monogastric (pig, poultry, human) is well understood and documented but in the transition period of dairy cows is still under investigation. The reviewed studies suggested that, considering RPC supplementation levels for best responses were noted to be 12–20 g/d of RPC in cows’ ration, but the recommended doses during transition period is still unclear. Therefore, the next step is to determine how much RPC should be given at transition period to overcome its related problems and maintain performance of dairy cows. Further epigenetic mechanism and omics technology might be an effective strategy to further understand choline, folate and methionine interaction and their significant functions during transition period.

## Abbreviations

AA: amino acid
ACho: Cho, acetylcholine
Bet: betaine
BHBA: β hydroxybutyric acid
Cho: free choline
GPCho: glycerol phosphorus choline
LPC: lysophosphatidylcholine
Met: methionine
NEFA: non-esterified fatty acids
PC: phosphatidylcholine
PCho: phosphorus choline
ROS: reactive oxygen species
RPC: rumen protected choline
SAM: *S*-adenosylmethionine
SM: sphingomyelin
T-AOC: total antioxidant capacity
TBIL: total plasma bilirubin
TG: triglyceride RPM: rumen protected methionine
VLDL: very low-density lipoprotein
